# Regulation and Function of FOXC1 in Osteoblasts

**DOI:** 10.3390/jdb11030038

**Published:** 2023-09-19

**Authors:** Sarocha Suthon, Jianjian Lin, Rachel S. Perkins, Gustavo A. Miranda-Carboni, Susan A. Krum

**Affiliations:** 1 Department of Orthopaedic Surgery and Biomedical Engineering, University of Tennessee Health Science Center, Memphis, TN 38163, USA; 2 Department of Medicine, University of Tennessee Health Science Center, Memphis, TN 38163, USA; 3 Center for Cancer Research, University of Tennessee Health Science Center, Memphis, TN 38163, USA

**Keywords:** FOXC1, GATA4, osteoblast, estrogen

## Abstract

Estrogens, which bind to estrogen receptor alpha (ERα), are important for proper bone mineral density. When women go through menopause, estrogen levels decrease, and there is a decrease in bone quality, along with an increased risk for fractures. We previously identified an enhancer near *FOXC1* as the most significantly enriched binding site for estrogen receptor alpha (ERα) in osteoblasts. FOXC1 is a transcription factor belonging to a large group of proteins known as forkhead box genes and is an important regulator of bone formation. Here, we demonstrate that 17β-estradiol (E2) increases the mRNA and protein levels of FOXC1 in primary mouse and human osteoblasts. GATA4 is a pioneer factor for ERα and it is also recruited to enhancers near *Foxc1*. Knockdown of *Gata4* in mouse osteoblasts in vitro decreases *Foxc1* expression as does knockout of *Gata4* in vivo. Functionally, GATA4 and FOXC1 interact and regulate osteoblast proteins such as RUNX2, as demonstrated by ChIP-reChIP and luciferase assays. The most enriched motif in GATA4 binding sites from ChIP-seq is for *FOXC1*, supporting the notion that GATA4 and FOXC1 cooperate in regulating osteoblast differentiation. Together, these data demonstrate the interactions of the transcription factors ERα, GATA4, and FOXC1 to regulate each other’s expression and other osteoblast differentiation genes.

## 1. Introduction

Transcription factors bind to enhancers and promoters to regulate gene expression. Tissue-specific gene regulation is controlled in part by the spatiotemporal and combinatorial binding of transcription factors. There are many known transcription factors in bone, including RUNX2, estrogen receptor alpha (ERα), GATA4, and FOXC1. Herein, we describe the transcriptional regulation of Foxc1 and Runx2 in bone that is mediated by ERα, GATA4, and FOXC1.

Estrogens are important for maintaining bone mineral density in both mice and humans via estrogen receptor alpha (ERα) [1]. Estrogen signaling regulates osteoblasts, osteoclasts, and the immune system to maintain bone mass. Specifically in osteoblasts, ERα inhibits apoptosis and induces the expression of differentiation genes such as BMP2, osteoprotegerin (OPG), and alkaline phosphatase [2,3].

GATA transcription factors, so named because they bind to the consensus DNA sequence (A/T)GATA(A/G), have been identified as important regulators of tissue-specific gene expression during development. GATA4 is necessary for heart, liver, and osteoblast differentiation, among other tissues [4,5]. Bone-specific deletion of GATA4 leads to embryonic lethality [3]. GATA4 recruits ERα to estrogen-regulated genes including alkaline phosphatase [6]. In addition, there are estrogen-independent functions of GATA4. Knockout of *Gata4* in osteoblasts using the *Prx1*, *Runx2*, or OCN (*Bglap*) promoters driving Cre-recombinase leads to trabecular bone loss [5,7,8]. GATA4 regulates many osteoblast differentiation genes, including *Runx2*, bone sialoprotein, and osteocalcin [3].

A pioneer factor is a protein that can bind to the genome in sites of condensed chromatin, leading to nucleosome remodeling and the opening of chromatin, the recruitment of additional transcription factors, and the activation of transcription [9]. GATA4 has been shown to be a pioneer factor in the liver, heart, and osteoblasts [6,10,11]. In the liver, this activity depends on FOXA proteins for stable nucleosome binding [11], as FOXA proteins can bind to closed chromatin and displace histones, leading to open and active enhancers. The binding of FOXA proteins to the DNA allows for GATA4 recruitment in liver cells [11]. However, FOXA1 is not expressed in osteoblasts [12], suggesting a role for a different forkhead protein to recruit GATA4 to chromatin in osteoblasts.

There are 50 human and 44 mouse forkhead proteins, categorized from FoxA to FoxS [13]. FOXC1 has been shown to be important for skeletal development. Mutations in *FOXC1* in humans cause Axenfeld–Rieger Syndrome, characterized by ocular defects but also skeletal abnormalities [14]. A spontaneous loss-of-FOXC1-function mutant mouse (named Foxc1^ch/ch^ because it has congenital hydrocephalus) dies at birth and has skull and axial and appendicular bone defects [15,16], demonstrating a role for FOXC1 in intramembranous and endochondral bone formation. Chondrocyte-specific deletion of *Foxc1* and *Foxc2* leads to disrupted endochondral ossification and skeletal dysplasia [17]. Mechanistically, FOXC1 has been shown to regulate osteoblast differentiation by regulating *Runx2,* the master transcription factor for osteoblastogenesis [18,19].

For the first time, we identified key regulators of *Foxc1* regulation and functions for FOXC1 in osteoblasts. Importantly, GATA4 and FOXC1 interact in osteoblasts to directly regulate *Runx2*.

## 2. Material and Methods

### 2.1. Mice

Animal experiments were approved by the Institutional Animal Care and Use Committee at the University of Tennessee Health Science Center. Animals were maintained in a specific pathogen-free environment at 20–26 °C with a relative humidity of 30–70% and a 12 h light/dark cycle. Commercial rodent chow (LM-485, Teklad, Madison, WI, USA) and drinking water were available ad libitum.

GATA4-Flag-biotin mice (Flag-bio, Gt(ROSA)26Sortm1(birA)Mejr Gata4tm3.1 Wtp/J) [20] were obtained from Jackson Labs (stock #018121). GATA4 Prx-cKO mice were previously described [7].

Six-week-old female BALB/c mice were sham-operated or ovariectomized and then treated with 50 mg/kg body weight of E2 or sesame oil (as a control) by intraperitoneal injection for 24 h.

ERα, ERβ, and ERαβ heterozygous mice were kindly provided by Dr. Pierre Chambon [21]. ERα^+/−^ERβ^+/−^ mice were bred to produce WT, ERαKO, and ERαβKO littermates.

Mouse calvarial osteoblasts were isolated from 2-day-old CD1 or Flag-bio mice by sequential collagenase digestion [22]. The cells were incubated for 40 min in α-MEM with 1.0 mg/mL collagenase P and 1.25% trypsin at 37 °C. The cells were then washed in α-MEM and then incubated in α-MEM-1.0 mg/mL collagenase P-1.25% trypsin for 1 h at 37 °C. Collagenase digestion was stopped by the addition of complete α-MEM media containing 10% FBS. The cells from the second digest were obtained and allowed to proliferate in α-MEM media containing 10% FBS. Differentiation was induced in α-MEM media containing 5 mM β-glycerophosphate and 100 mg/mL ascorbic acid (mineralization medium) for 14 days.

### 2.2. Human Cells

The human study was approved by the Institutional Review Board at the University of Tennessee Health Science Center, and all individuals provided informed written consent before participation. Primary human osteoblasts were isolated from the trabecular bone in the femoral or humeral heads of individuals who underwent total joint replacement surgery, as described previously [23].

### 2.3. Cell Lines

Human osteosarcoma U2OS-ERα cells were kindly provided by Dr. Thomas Spelsberg and were maintained as described [24]. ERα expression was induced by treatment with 100 ng/mL doxycycline (DOX; Sigma-Aldrich Co., St. Louis, MO, USA) for 24 h after culture in phenol-red-free media containing 5% CDT-FBS for three days. Cells were treated with 10 nM E2 or vehicle control (ethanol, EtOH). The cell line was verified each year by STR profiling and tested for mycoplasma.

### 2.4. shGATA4

Lentivirus shC (a short hairpin that does not recognize any mammalian DNA) and shGATA4 were purchased from Sigma Aldrich. Two different shRNAs from the RNAi Consortium (TRC) in the pLKO vector were used to knockdown mouse *Gata4* (TRCN0000095215:

CCGGCCCAATCTCGATATGTTTGATCTCGAGATCAAACATATCGAGATTGGGTTTTTG and TRCN0000095217: CCGGCATCTCCTGTCACTCAGACATCTCGAGATGTCTGAGTGACAGGAGATGTTTTTG). The knockdown of *Gata4* was confirmed by qPCR.

### 2.5. RNA and qPCR

RNA and qPCR were performed as previously described [8]. The specific primers used for SYBR Green assays are listed in Supplemental Table S1. For analysis of the cDNA data, the values were normalized to β-actin (*Actb*) values.

### 2.6. Immunoprecipitation and Immunoblotting

Immunoprecipitation (IP) reactions were conducted on whole-cell lysates prepared in standard RIPA buffer extracted from calvarial osteoblasts. IPs were performed with antibodies to GATA4 (Clone G-4, Santa Cruz Biotechnology, Dallas, TX, USA) or normal mouse IgG, along with Protein G magnetic beads (Invitrogen, ThermoFisher, Waltham, MA, USA). Immunoblotting was performed with antibodies to FOXC1 (Cell Signaling Technology, clone D8A6, Danvers, MA, USA) and β-actin (Cell Signaling Technology, clone 8H10D10). Whole blots are shown in Supplemental Figure S1.

### 2.7. Chromatin Immunoprecipitation (ChIP)

ChIP was performed as previously described for U2OS cells [6] and Flag-biotin-tagged GATA4 [5]. ChIP-sequencing was previously described [8]. Motif analysis was performed using Meme Suite [25]. FOXC1 was immunoprecipitated with a goat polyclonal antibody (Abcam, ab5079). The specific primers for SYBR Green assays used for qPCR are listed in Supplemental Table S1. All data were compared to the percent input and a negative control region [8].

### 2.8. ChIP-reChIP

ChIP was performed as described above, except that after the first overnight incubation with the first antibody, the magnetic beads were washed 3 times with PBS/BSA and then resuspended in TE with 10 mM DTT for 30 min. The eluate was resuspended in ChIP dilution buffer, and the second antibody was added overnight.

### 2.9. Immunohistochemistry (IHC)

Formalin-fixed, paraffin-embedded samples were processed through standard deparaffinization protocols. Antigen retrieval was performed by placing the slides in 90 °C citrate buffer and leaving them at room temperature until the temperature reached 55 °C. The tissue was then incubated in blocking buffer (5% normal goat serum and 2.5% bovine serum albumin [BSA] in PBS at pH 7.5) for 30 min. Anti-FOXC1 (LSBio catalog #LS-B1800, Shirley, MA, USA) antibodies were incubated overnight at 4 °C in a humidified chamber, followed by the DAKO Envision Visualization system (Agilent, Santa Clara, CA, USA) and counterstaining with hematoxylin. Immunohistochemistry samples were scored without bias by three independent people using a bone-specific immune reactive scoring index of 0 (negative) -12 (strongly positive), multiplying the percent of positive cells by the intensity of staining [26].

### 2.10. Luciferase Assay

U2OS osteosarcoma cells were plated in 24-well tissue culture dishes and transfected with the rat 0.6 kb *Runx2*-promoter-luciferase (a kind gift from Dr. Gary S. Stein) [27], pcDNA3-*Gata4* (a kind gift from Dr. Michael Parmacek [28]), and pRL-SV40 (Promega, Madison, WI, USA) as a transfection control. pcDNA3.1-DYK-*Foxc1* was purchased from GenScript.

### 2.11. Cistrome Data Browser

The Cistrome Data Browser [29,30] was used to search for factors 100 kb from the transcriptional start site of *Foxc1*. The datasets that were used are listed below in the Data Availability section.

### 2.12. FOXC1 Expression in Osteoporotic Patients

The expression of *FOXC1* in Human Mesenchymal Stem Cells from old, age-matched osteoporotic and non-osteoporotic individuals was obtained in the GEO dataset GSE35959 [31].

### 2.13. Statistical Analysis

All experiments represent both biological and experimental triplicates. Unless otherwise stated, error bars represent the mean ± 1 standard deviation. Statistical analyses, including Student’s *t*-test, were performed using GraphPad Prism^®^ (version 9, Boston, MA, USA) software.

## 3. Results

### 3.1. The Transcriptional Regulation of Foxc1

**Figure 1:**
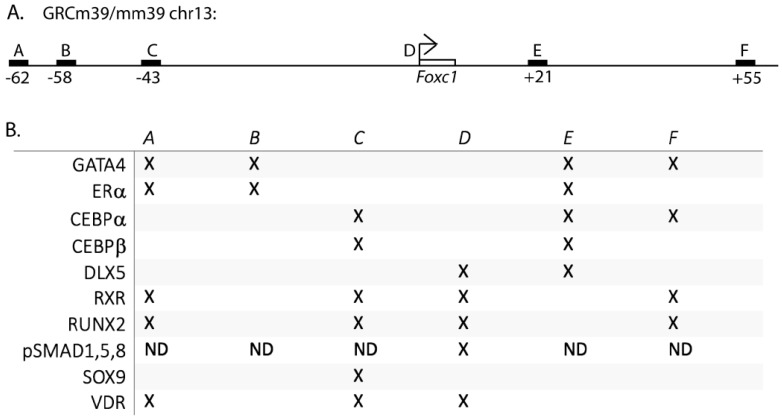
Transcription factors regulating *Foxc1*. (**A**) Schematic of enhancers near *Foxc1*. (**B**) “X” indicates the presence of the transcription factor at the indicated enhancer or promoter by ChIP-sequencing and/or ChIP-qPCR. ND = not determined.

To search for estrogen-regulated genes in osteoblasts, we analyzed our ERα binding sites in U2OS-ERα cells after 45 min. of 17β-estradiol (E2) treatment [6]. U2OS-ERα cells are an osteosarcoma cell line that has an inducible expression of ERα. Overexpression of ERα in U2OS cells leads to an osteoblast phenotype that serves as a model for normal osteoblasts [12,23,24,32]. The ERα binding site with the highest enrichment was near *FOXC1* and FOXC1 has been shown to be important for osteoblast biology [18,19,33]. Therefore, we first searched the literature for transcription factors that could regulate *FOXC1* in bone cells. All of the papers investigated murine *Foxc1* regulation. SOX9 was shown to bind 43 kb upstream of *Foxc1* (site C, Figure 1A,B) by ChIP-sequencing in chondrocytes [17]. In addition, phosphorylated SMAD1,5,9 was shown to bind 839 to 384 bp upstream of the *Foxc1* transcriptional start site [33] (site D, Figure 1A,B). (ChIP-sequencing was not performed to look for additional sites for pSMAD1,5,9 recruitment).

Next, we retrospectively searched our ERα (translated to the murine genome) and GATA4 ChIP-sequencing data in combination with publicly available ChIP-seq data obtained from the Cistrome Data Browser for additional transcription factors that bind near *Foxc1* in MSCs, osteoblasts, and chondrocytes. We identified additional enhancers near *Foxc1* bound by CEBPα, CEBPβ, DLX5, RUNX2, RXR, and VDR (Figure 1A,B). Each of these proteins has been shown to have important roles in bone development. Interestingly, the location and number of binding sites are different for each transcription factor, demonstrating complex regulation involving over 100 kb.

### 3.2. Estrogen Regulates Foxc1 in Osteoblasts

**Figure 2:**
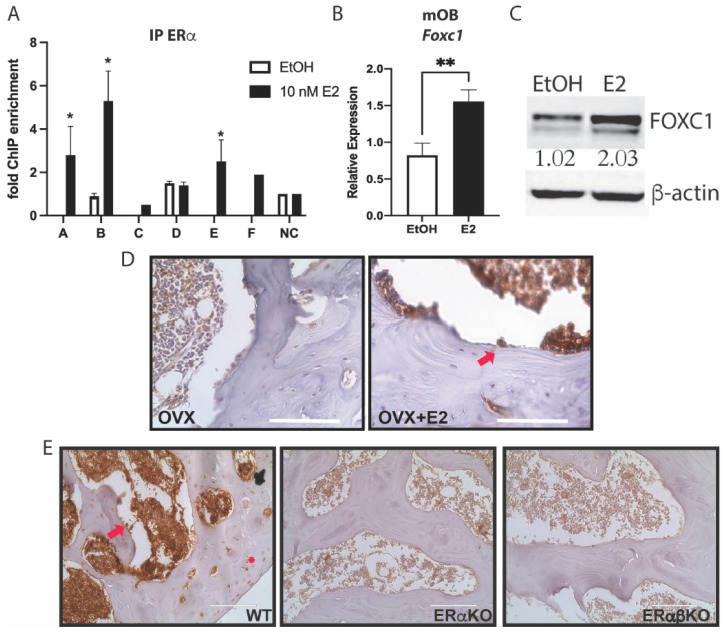
ERα regulates *Foxc1*. (**A**) Primary calvarial osteoblasts were treated with ethanol (EtOH) as a vehicle control or 10 nM E2 for 45 min before being fixed with 4% PFA. ChIP was performed with an antibody to ERα. qPCR was performed at the indicated regions near *Foxc1* or a negative control (NC) region. (**B**) Primary calvarial osteoblasts were treated with EtOH or 10 nM E2 for 24 h. RNA was obtained, and qPCR was performed with primers for *Foxc1* and normalized to *Actb* mRNA. (**C**) Primary calvarial osteoblasts were treated with 10 nM E2 for 24 h. Protein was obtained, and immunoblotting was performed with antibodies to FOXC1 and β-actin. The bands were quantified and normalized to β-actin and are displayed under the FOXC1 band. (**D**) Immunohistochemistry was performed with an antibody to FOXC1 (brown) on femurs from ovariectomized mice treated with or without 50 mg/kg body weight of E2 for 24 h. The slides were co-stained with hematoxylin. The arrow indicates an example of a FOXC1+ osteoblast. Bar = 100 μm. (**E**) Immunohistochemistry was performed with an antibody to FOXC1 (brown) on femurs from wild-type, ERαKO, and ERαβKO mice and co-stained with hematoxylin. The arrow indicates an example of a FOXC1+ osteoblast. * indicates an example of a FOXC1+ chondrocyte. Bar = 100 μm. * = *p* value < 0.05, ** = *p* value < 0.01.

To verify the binding sites for ERα near *Foxc1*, primary mouse calvarial osteoblasts were treated with 10 nM E2 for 45 min., and ChIP was performed. qPCR primers were designed for these predicted enhancers and the promoter of *Foxc1*. Indeed, after E2 treatment, ERα was enriched at enhancers A, B, and E (Figure 2A), compared to untreated cells or a negative control (NC) genomic region.

To determine if E2 induced expression of *Foxc1* after ERα binding, calvarial osteoblasts were treated with 10 nM E2 for 24 h. RNA and protein were obtained and analyzed for the expression of *Foxc1*. qPCR (Figure 2B) and immunoblotting (Figure 2C) both demonstrate that E2 induced a 2-fold increase in *Foxc1* expression.

Next, we analyzed the effect of E2 on FOXC1 in bone in vivo. Mice were ovariectomized (OVX) or sham-operated and then treated with vehicle or E2 for 24 h. Immunohistochemistry was performed with an antibody to FOXC1. FOXC1 is increased in the E2-treated osteoblasts found along the trabecular bone (Figure 2D). To demonstrate that ERα is necessary for the regulation of *Foxc1*, immunohistochemistry for FOXC1 was also performed on the femurs from WT, ERα knockout (ERαKO), and ERα/ERβ double knockout (ERαβKO) mice. FOXC1 staining was quantified by the number of positive cells and the intensity of staining. FOXC1 was markedly reduced in the ERαKO and ERαβKO mice (Figure 2E and Supplemental Figure S2).

Together, these experiments demonstrate that E2 and ERα directly up-regulate *Foxc1* expression by binding to *Foxc1* enhancers.

### 3.3. GATA4 Regulates Foxc1 Expression

**Figure 3:**
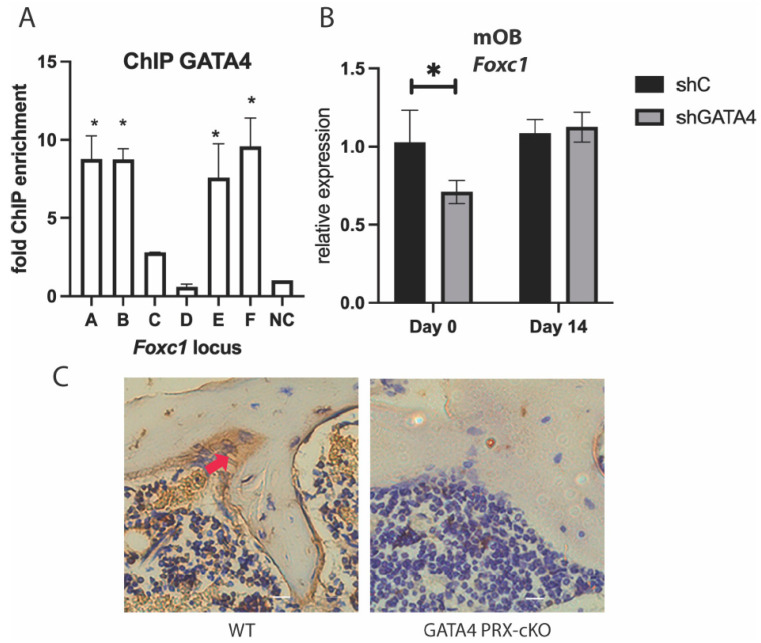
GATA4 regulates *Foxc1*. (**A**) Primary calvarial osteoblasts from GATA4 Flag-bio mice or WT CD1 mice were fixed, and ChIP was performed with streptavidin beads. qPCR was performed at a region upstream and downstream of the *Foxc1* gene. (**B**) Primary calvarial osteoblasts were exposed to lentivirus encoding shRNA for *Gata4* or a negative control. RNA was obtained, and qPCR was performed with primers for *Foxc1* and normalized to *Actb* mRNA. (**C**) Immunohistochemistry on femurs from wild-type or GATA4 PRX1-cKO was performed with an antibody to FOXC1 (brown) and co-stained with hematoxylin. The arrow indicates an example of a FOXC1+ osteoblast. * = *p* value < 0.05.

We have previously demonstrated that GATA4 is a pioneer for ERα in osteoblasts [6]. To determine if GATA4 also regulates *Foxc1*, ChIP was performed to detect GATA4 at the enhancers near *Foxc1*. Because of inferior antibodies to GATA4, a mouse with GATA4 tagged with the Flag epitope and a biotinylation site at the endogenous locus was created (GATA4 Flag-bio mice) and has been successful for GATA4 ChIP [5,7,8]. Calvariae from these mice were obtained, and ChIP was performed with streptavidin beads. GATA4 was enriched at enhancers A, B, E, and F in the GATA4 Flag-bio mice (Figure 3A). GATA4 has ERα-independent functions [3], as evidenced by enhancer F, which is bound by GATA4 but not ERα.

To further demonstrate that GATA4 regulates *Foxc1*, wild-type calvarial cells were cultured and knockdown of *Gata4* was performed with lentiviral shRNA to *Gata4* [3]. Knockdown of *Gata4* correlated with a decrease in *Foxc1* mRNA at day 0 of differentiation, but not at day 14, (Figure 3B), which is consistent with a role for GATA4 early in differentiation [3]. Conditional knockout (cKO) of *Gata4* in osteoblast progenitors (using PRX1-CRE) causes osteopenia [7]. Immunohistochemistry with an antibody to FOXC1 revealed a significant decrease in FOXC1 protein in the cKO femurs (Figure 3C and Supplemental Figure S2). WT mice express FOXC1 along the trabecular bone and in the bone marrow, whereas the *Gata4* cKO mice have lost nearly all expression of FOXC1.

Together, these experiments demonstrate that GATA4 in vivo and in vitro directly up-regulates *Foxc1* expression by binding to *Foxc1* enhancers.

### 3.4. GATA4 and FOXC1 Interact in Osteoblasts to Regulate Osteoblast Differentiation

**Figure 4:**
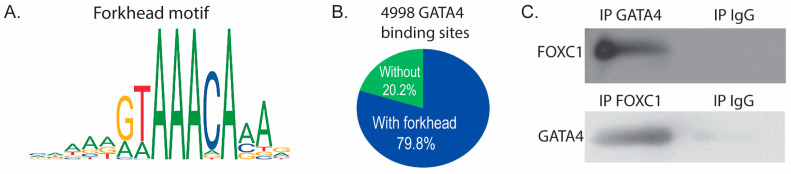
FOXC1 and GATA4 interact to regulate bone genes. (**A**) ChIP sequencing for GATA4 was performed on calvarial osteoblasts obtained from GATA4 Flag-bio mice. GATA4 binding sites were analyzed for enriched motifs, which included the depicted motif for FOXC1. (**B**) 79.8% of the GATA4 binding sites contained the motif MA0481.1 (the motif depicted in part (**A**)). (**C**) Equal amounts of wild-type calvarial osteoblast protein were immunoprecipitated with FOXC1, GATA4, or normal IgG. Then immunoblots were performed to detect FOXC1 or GATA4.

Analysis of our GATA4 ChIP-seq data [7] using Analysis of Enriched Motifs (AME) [34] software revealed that the most highly enriched motif (*p* value < 3.95 × 10^−100^) in the 5983 identified GATA4 binding sites is a canonical forkhead motif. Simple Enrichment Analysis (SEA) [25] also identified a forkhead motif (MA0481.1, *p* value < 3.15 × 10^−15^, Figure 4A). 79.8% of the GATA4 binding sites contained the motif MA0481.1 (Figure 4B), suggesting that FOXC1 binds with or near GATA4 to regulate osteoblast-specific genes. Because the GATA4 ChIP-seq data predicts a forkhead protein/FOXC1 at the same binding sites, we sought to determine if GATA4 and FOXC1 physically interact through direct or indirect protein–protein interactions. Co-immunoprecipitations were performed with an antibody to GATA4, FOXC1, or normal IgG and demonstrated that GATA4 and FOXC1 interact in calvarial osteoblasts (Figure 4C).

**Figure 5:**
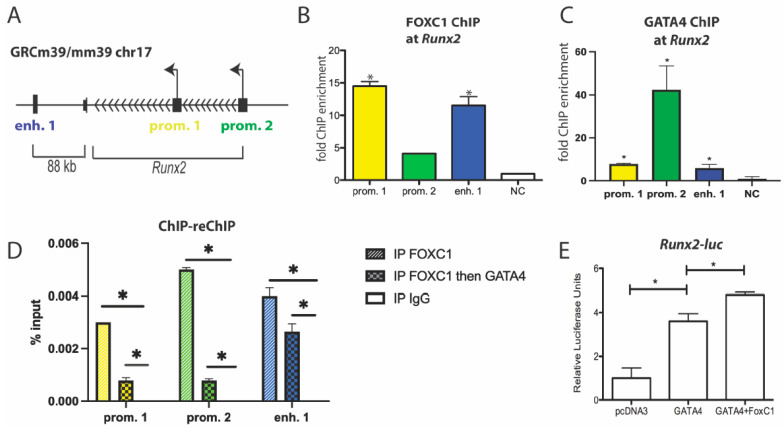
GATA4 and FOXC1 regulate *Runx2.* (**A**) Schematic diagram of the mouse *Runx2* genomic locus. The arrowheads indicate the direction of gene transcription. The transcriptional start sites are shown by the arrow. GATA4 binding sites are denoted at the promoters and enhancer 1. (**B**) ChIP was performed in primary calvarial osteoblasts with an antibody to FOXC1. qPCR was performed with primers for the indicated regions near *Runx2* or a negative control genomic region. (**C**) ChIP was performed using calvaria from GATA4 Flag-bio mice or wild-type (WT) mice and streptavidin beads to detect GATA4. qPCR was performed with primers for the indicated regions near *Runx2* or a negative control genomic region. (**D**) ChIP was performed on whole-cell lysate from U2OS cells with an antibody to FOXC1. ChIP-reChIP was performed first with an antibody to FOXC1 and then GATA4. IgG was used as a negative control. (**E**) The *Runx2*-promoter luciferase construct was transfected into U2OS cells along with pcDNA3-GATA4 or FOXC1, where indicated. Luciferase values were normalized to Renilla luciferase. *n* = 3. * = *p* value < 0.05.

We have previously shown that GATA4 binds to both promoters of *Runx2* and to a downstream enhancer (Figure 5A) to regulate its expression and osteoblast differentiation [5]. In primary osteoblast cells, FOXC1 and GATA4 are recruited to these genomic regions, as detected by ChIP on the *Runx2* enhancer 1 and on both promoters 1 and 2 (Figure 5B,C). ChIP-reChIP showed that FOXC1 and GATA4 are at the *Runx2* promoters and an enhancer at the same time (Figure 5D). In addition, GATA4 enhances the transcription of a *Runx2*-luciferase plasmid containing the osteoblast-specific P1 promoter [35], and the addition of FOXC1 enhances this transcriptional regulation (*p* < 0.01) (Figure 5E). These results support the notion that FOXC1 is directly enhancing GATA4 activation of *Runx2* expression.

### 3.5. Clinical Relevance of FOXC1 and Osteoporosis

**Figure 6:**
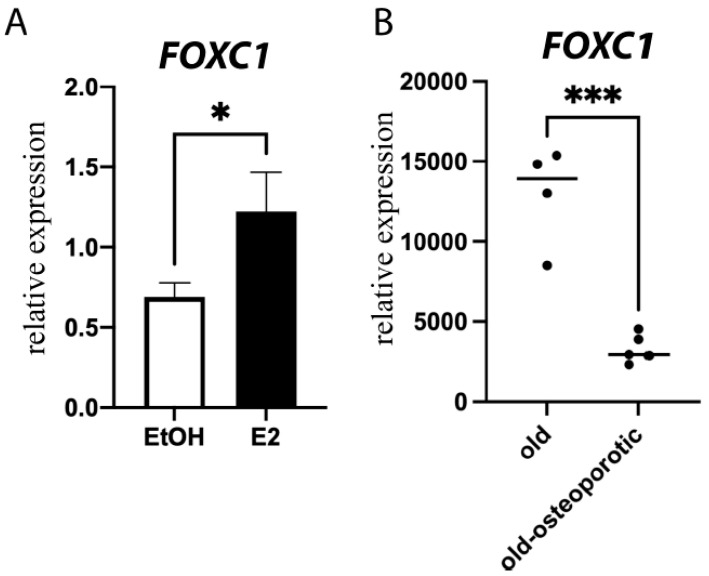
FOXC1 in human cells. (**A**) Primary human osteoblasts were treated with vehicle control (Ethanol, EtOH) or 10 nM E2 for 24 h. RNA was obtained, and qPCR was performed for *FOXC1* and normalized to *ACTB*. (**B**) GEO dataset GSE35959 was analyzed for the expression of *FOXC1* in age-matched elderly individuals with or without osteoporosis. * = *p* value < 0.05; *** = *p* value < 0.001.

FOXC1 has been mostly studied in murine models of bone, and little work has been conducted in human models to determine whether it is clinically important. However, we show that E2 induces *FOXC1* expression not only in murine calvarial osteoblasts (Figure 2) but also in primary human osteoblasts (Figure 6A) and the U2OS-ERα model system (Supplemental Figure S3).

Because we saw a decrease in FOXC1 in ovariectomized mice, we sought to determine if there is a decrease in FOXC1 in osteoporosis. Publicly available expression arrays of mesenchymal stem cells from osteoporotic individuals demonstrate significantly lower expression of *FOXC1* in comparison with age-matched elderly (79–94 years old) individuals (Figure 6B). These data suggest that *FOXC1* expression is clinically relevant for osteoporosis.

## 4. Discussion

ERα, GATA4, and FOXC1 are important transcription factors in osteoblast differentiation. Here we show that the transcription factors ERα and GATA4, along with SOX9, pSMAD1,5,9, CEBPα, CEBPβ, DLX5, RUNX2, RXR, and VDR, regulate *Foxc1* expression. Furthermore, we demonstrated that GATA4 and FOXC1 physically interact to regulate the master osteoblast transcription factor *Runx2*.

FOXC1 is critical to bone development as it controls endochondral and intramembranous ossification [14,15,16,17]. Loss-of-function of FOXC1 in mice and humans leads to developmental abnormalities in the appendicular and axial skeletons. Mechanistically, FOXC1 has been shown to regulate *Runx2* [18], β-catenin [36,37], and *Msx2* [19], all important regulators of skeletogenesis. In osteoblasts, FOXC1 binds to the *Runx2* promoter and activates the expression of *Runx2* in response to intermittent parathyroid hormone in MC3T3-E1 cells [18]. FOX proteins can function as either classical transcription factors, pioneer factors, or cofactors [38]. We show that FOXC1 interacts with GATA4 and upregulates *Runx2* by binding to an enhancer and its promoters in mouse osteoblasts. FOXC1 also binds to the *DKK1* promoter to suppress expression [36], while GATA4 is also recruited to the *DKK1* promoter and enhancer to activate WNT signaling in osteoblasts [7]. Moreover, we find that 79.8% of the GATA4 binding sites contained the motif MA0481.1, a forkhead recognition site. This suggests a coregulation pattern between FOXC1 and GATA4 in osteoblasts. Other osteogenic markers such as *Osx*, *Alp*, and *Bglap (osteocalcin)* are upregulated by FOXC1 [18,19]. Thus, ChIP-sequencing of FOXC1 would provide many additional gene targets in osteoblast differentiation. An analysis of histone modifications and the timing of FOXC1 and GATA4 binding would help determine if they are pioneer factors.

GATA4 and ERα show synchronized actions on osteoblast differentiation by activating the expression of *RUNX2*, *ALPL*, and *BGLAP* [3,6]. GATA4 and ERα interact and bind at either estrogen response elements or GATA binding sites for the cis-regulation of many genes [32]. We elucidate that GATA4 and ERα control the expression of *Foxc1* by binding to enhancers in mouse osteoblasts. Mechanistically, activation of ERα increases its binding and the expression of *FOXC1*, while *GATA4* knockdown abolishes the binding of ERα at the *FOXC1* enhancer in U2OS-ERα cells [6]. We show multiple transcription factors, in addition to ERα and GATA4 bind upstream and downstream of *Foxc1*. Binding alone is not sufficient for transcriptional regulation [12], so functional experiments could be done to show up- or down-regulation of *Foxc1* mRNA. Furthermore, chromatin conformation assays would reveal additional complexity of the *Foxc1* locus.

Osteosarcoma is a bone cancer that can be caused by osteoblast-committed cells with differentiation defects [39]. *FOXC1* shows high expression in osteosarcoma and promotes cancer progression and metastasis [37,40]. *RUNX2* also has high expression in osteosarcoma and correlates with metastasis and poor survival [41,42]. This suggests a correlation between FOXC1 and RUNX2 in osteosarcoma. Thus, understanding the function of FOXC1 in normal osteoblast differentiation may help find the role of FOXC1 in osteosarcoma and osteoporosis.

In conclusion, we show that GATA4 and FOXC1 directly promote *Runx2* expression by interacting with and binding to the *Runx2* enhancer and its two promoters. Furthermore, ERα and GATA4 control the expression of *Foxc1*, in addition to other transcription factors and cofactors.

## Data Availability

The following data was obtained from NCBI GEO: The expression of *FOXC1* in Human Mesenchymal Stem Cells from old age-matched osteoporotic and not-osteoporotic individuals was obtained in the GEO dataset GSE35959.
ChIP-seq
ERαGSE28918CEBPαGSM2104127CEBPβGSM2104228DLX5GSM1976254RXRGSM2104118RUNX2GSM2104159SOX9GSM826703VDRGSM2104116

## Supplementary Materials

The following supporting information can be downloaded at: https://www.mdpi.com/article/10.3390/jdb11030038/s1, Figure S1: Whole immunoblots from Figure 2 and Figure 4; Figure S2: Immunoreactivity scoring; Figure S3: Foxc1 regulation by E2 in human cells; Table S1: Primers.
