# Supplementary material for: Regulation and Function of FOXC1 in Osteoblasts
Source: J Dev Biol. 2023 Sep 19;11(3):38. doi: 10.3390/jdb11030038 (PMC10531946; doi:10.3390/jdb11030038)
Supplement: Supplementary file 1 [file jdb-11-00038-s001.zip › jdb-2588861-supplementary.pdf]

Journal of Developmental Biology

MDPI

Supplemental Figure S1:

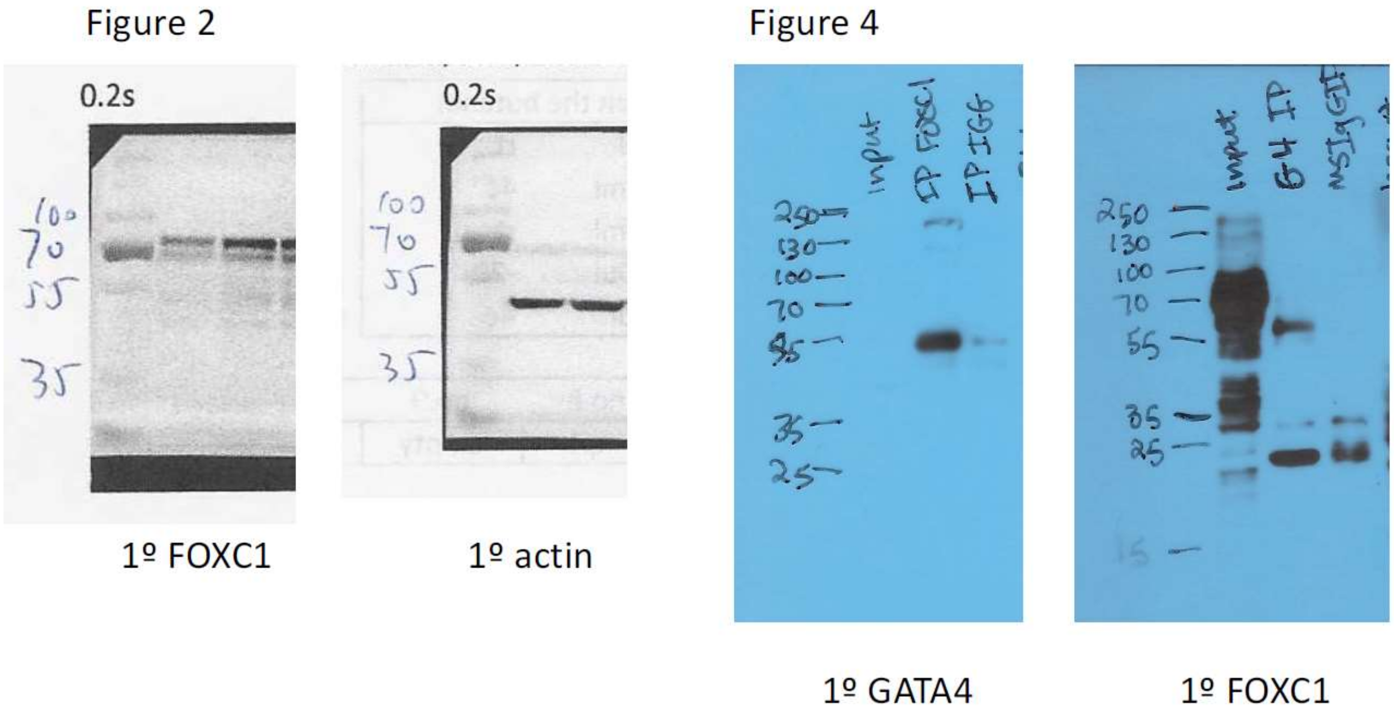

Figure S1: Whole immunoblots from Figures 2 and 4.

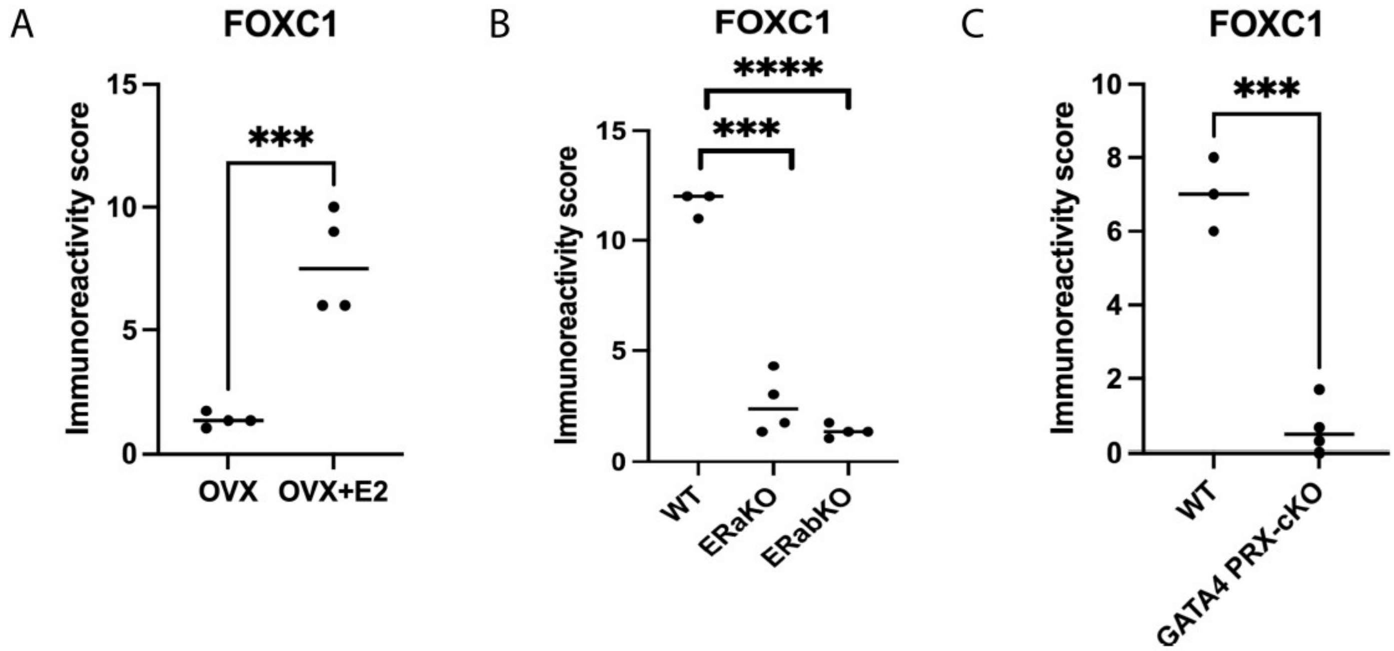

Supplemental Figure S2: (A) Immunoreactivity scoring of IHC in Figure 2D. (B) Immunoreactivity scor-ing of IHC in Figure 2E. (C) Immunoreactivity scoring of IHC in Figure 3.

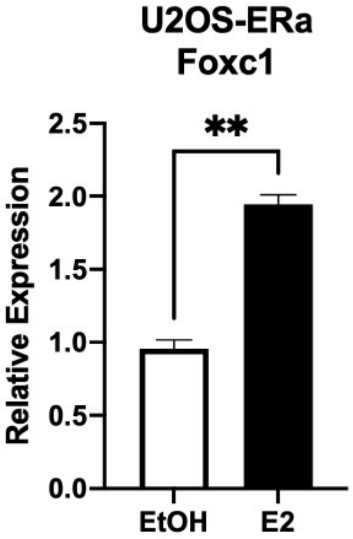

Supplemental Figure S3: *Foxc1* regulation by E2 in human cells. U2OS-ERα cells were treated with vehicle control (Ethanol, EtOH) or 10 nM E2 for 24 hours. RNA was obtained and qPCR was performed for *FOXC1* and normalized to *ACTB*.

Supplemental Table S1.

Primers used for ChIP	Sequence	Species
Runx2 prom 1	CCCCCAAAGTGGGGAGTGGC	mouse
	TGCTTGCTGGGAAAGGGGGA	
Runx2 prom 2	ACCTTCTGAATGCCAGGAAGGCCT	mouse
	TGGGACTGCCTACCACTGTGG	
Runx2 enh	TCCTGGACCAGAGCCACGTT	mouse
	GCAACTCCCTGGATGCCCTGG	
NC	GTGGCTGCTCTTCGCTGCCA	mouse
	TGCTGCCACCTGGCCTTTGG	
Foxc1 A	ACTGCTGCGAAAGGTCTAGG	mouse
	GAGCAACAGACCACGCAATC	
Foxc1 B	AAGCCCCTAGCTGGTTTCAT	mouse
	GGAGTGGGGTGTCTGTCAGT	
Foxc1 C	TCGCAAGCTGGTCGTAATTC	mouse
	CATACCTAGCCCAGCCTTTGT	
Foxc1 D	TCATTCACAGGCGTCTCTCG	mouse
	TTTTGAGCATCCGTCACCCA	
Foxc1 E	GTGGCATAAACCCCGAGGAA	mouse
	GAGGCCTCTGAAGCCGATAC	
Foxc1 F	CACCGTCAAACTCAAGCAGC	mouse
	TACATGAGGGGCTCTGGGAA	
Primers for cDNA	Sequence	Species
Actin (Actb)	TGGGACTGCCTACCACTGTGG	mouse
	TCCTGGACCAGAGCCACGTT	
Foxc1		mouse
Actin (ACTB)	TCCTGGACCAGAGCCACGTT	human
FOXC1	GTGGCTGCTCTTCGCTGCCA	human
	TGCTGCCACCTGGCCTTTGG	
